# Methotrexate-induced acute neurotoxicity in patients with osteosarcoma: a case report

**DOI:** 10.1186/s13256-025-05500-y

**Published:** 2025-09-30

**Authors:** Olivia L. Makos, Nicole A. Shonka, Kealy M. Marth, Shawna L. Stricker, Mark Keiper, Makayla E. Schissel

**Affiliations:** 1 Department of Internal Medicine, University of Nebraska Medical Center, 986840 Nebraska Medical Center, Omaha, NE 68198 USA; 2 Division of Oncology/Hematology, University of Nebraska Medical Center, Omaha, USA; 3 Department of Pharmacy, Nebraska Medicine, Omaha, USA; 4 Department of Radiology, University of Nebraska Medical Center, Omaha, USA; 5 Department of Biostatistics, University of Nebraska Medical Center, Omaha, USA

**Keywords:** Methotrexate, Neurotoxicity, Leukoencephalopathy, Rechallenge, Case report

## Abstract

**Background:**

Methotrexate is commonly used to treat osteosarcoma and acute lymphoblastic leukemia. Methotrexate can rarely cause neurotoxicity with a wide range of presentations including seizure, hemiparesis, dysarthria, dysphagia, and more. Acute neurotoxicity typically occurs within 2–14 days after methotrexate administration. The incidence of methotrexate-induced neurotoxicity, risk factors, treatments, and recurrence of neurotoxicity on methotrexate rechallenge all largely come from literature involving patients with acute lymphoblastic leukemia.

**Case presentation:**

We present a case of methotrexate-induced neurotoxicity and leukoencephalopathy in a 20-year-old Hispanic male with osteosarcoma who improved after treatment with dextromethorphan and aminophylline. To better understand methotrexate-induced neurotoxicity in patients with osteosarcoma specifically, we conducted a literature review of 16 cases, including ours.

**Conclusion:**

To the knowledge of these authors, this is the largest compilation of cases of methotrexate-induced neurotoxicity involving patients with osteosarcoma. There is no standard treatment for methotrexate-induced neurotoxicity. In our review we discuss dextromethorphan, aminophylline, and ketamine use in the treatment of methotrexate-induced neurotoxicity. Methotrexate is a crucial, first-line treatment for osteosarcoma and if safe, would be beneficial to continue even after acute neurotoxicity. Unfortunately, methotrexate is often discontinued after the first episode of neurotoxicity, owing to fear of recurrence on rechallenge. In our review, 5 of 16 patients were known to be rechallenged with methotrexate. None had recurrence of neurotoxicity with subsequent methotrexate treatment. While our study is limited by the number of cases, our findings suggest that methotrexate rechallenge in patients with osteosarcoma could be considered. Our review adds to the limited existing literature on patients with osteosarcoma with methotrexate-induced neurotoxicity and can aid in the understanding of the complicated pathophysiology, available treatments, and decision of whether to proceed with methotrexate rechallenge.

**Supplementary Information:**

The online version contains supplementary material available at 10.1186/s13256-025-05500-y.

## Background

Methotrexate (MTX) is a folate antimetabolite used to treat malignancies, and certain autoimmune conditions [1]. Specifically, high-dose MTX (HDM) with leucovorin rescue is commonly used to treat osteosarcoma and acute lymphoblastic leukemia (ALL). MTX can cause neurotoxicity with a wide range of presentations including headache, seizure, hemiparesis, dysarthria, dysphagia, and more [2]. Clinical symptoms of MTX-induced neurotoxicity are often associated with leukoencephalopathy, seen on imaging as transient diffuse white matter hyperintensities, classically in the centrum semiovale on T2-weighted and fluid-attenuated inversion recovery (FLAIR) magnetic resonance imaging (MRI). These changes may be unilateral, bilateral, or alternating between the two over the course of the disease. However, the key characteristic radiological marker is most appreciated on diffusion weighted imaging (DWI) MRI, which demonstrates regions of restricted diffusion across multiple vascular territories in the centrum semiovale, again either unilateral, bilateral, or alternating, that eventually disappear after symptom resolution. These DWI changes are thought to be reliable and early signs of acute methotrexate-related leukoencephalopathy [2–4]. It is important to note that not all symptomatic patients have leukoencephalopathy, and that leukoencephalopathy can also develop in asymptomatic patients receiving MTX. The clinical significance of these white matter changes is not known, and it is unclear whether patients with asymptomatic leukoencephalopathy are at higher risk of developing symptoms when exposed to additional MTX [5].

MTX-induced encephalopathy typically occurs within 2–14 days after MTX administration [6]. The incidence of MTX-induced neurotoxicity, risk factors, treatments, and recurrence of neurotoxicity on MTX rechallenge all largely comes from patients treated for ALL [1, 7]. We present a case of MTX-induced neurotoxicity and leukoencephalopathy in a young Hispanic adult with osteosarcoma who improved after treatment with dextromethorphan and aminophylline. To better understand MTX-induced neurotoxicity in patients with osteosarcoma specifically, we conducted a literature review of 16 cases, including ours. To the knowledge of these authors, this is the largest compilation of cases of MTX-induced neurotoxicity in osteosarcoma that exists.

## Case presentation

A 20-year-old Hispanic male with newly diagnosed stage IIB osteosarcoma of the left distal femur presented with slurred speech and right-sided hemiplegia 9 days after receiving his fourth cycle of methotrexate. Notably, he was receiving MTX, doxorubicin, and cisplatin (MAP therapy) per the AOST0331 protocol, which includes neoadjuvant doxorubicin/cisplatin every 3 weeks and MTX every fourth and fifth week. MTX blood levels throughout his fourth cycle were all within goal range and MTX blood level on the last day of cycle 4 was 0.07 mcmol/L (goal range < 0.1 mcmol/L). Interestingly, during the fourth cycle of MTX, he also received fosaprepitant for nausea, which is known to cause worse neurotoxicity when coadministered with ifosfamide, but has not been reported to worsen neurotoxicity when administered with MTX.

On the day of his presentation for right-sided hemiplegia and dysarthria, brain MRI with and without contrast showed three separate foci of restricted diffusion within the centrum semiovale without T2/FLAIR correlate or enhancement, consistent with MTX-induced leukoencphalopathy. MTX levels in the blood were undetectable at less than 0.05 mcmol/L and he was started on oral (PO) dextromethorphan (DM) 1 mg/kg twice daily (BID).

**Fig. 1:**
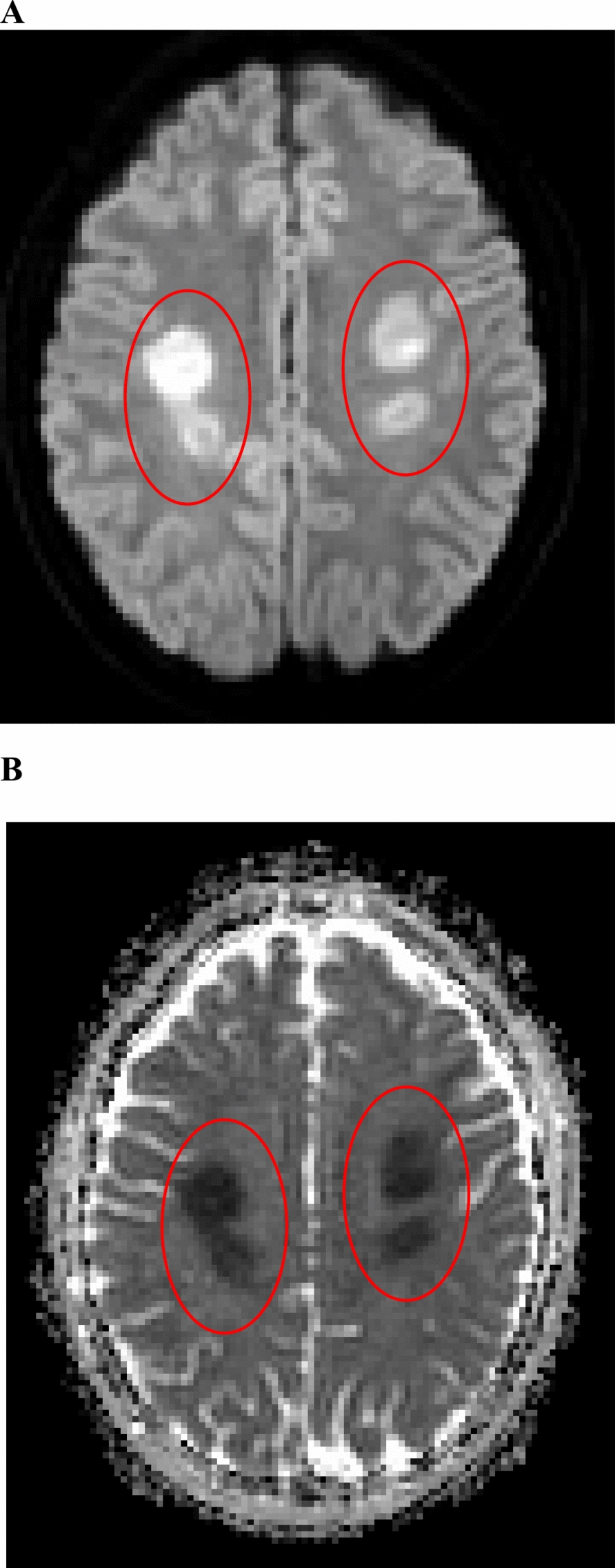
Magnetic resonance imaging of bilateral white matter changes with restricted diffusion (marked by the red circles) within the centrum semiovale on day 3 of hospitalization. **A** axial diffusion weighted imaging, (**B**) axial apparent diffusion coefficient map. The diffusion weighted image demonstrates increased signal, and the apparent diffusion coefficient map demonstrates corresponding decreased signal indicating restricted diffusion

The second day, he became nonverbal and somnolent. Left upper and lower extremity weakness, rigidity, clonus, and upgoing Babinski were also noted in addition to his initial right sided hemiplegia. Repeat brain magnetic resonance imaging (MRI) showed an increase in the size of the centrum semiovale diffusion restricting lesions, development of a new diffusion restriction within the right posterior centrum semiovale, and increased peripheral T2 FLAIR signal in the same regions. (Fig. 1) One dose of intravenous (IV) aminophylline 150 mg (2.5 mg/kg) in addition to PO DM 1 mg/kg BID.

The third day, he became more somnolent and dysphagic. IV aminophylline 100 mg every 6 hours was started and PO DM was increased to three times daily. On the fourth day of admission, his mental status began to improve. He fully returned to his former neurological baseline by day 13. He underwent below knee amputation for primary tumor resection and was discharged to inpatient rehabilitation. On outpatient follow-up visits he continued to be asymptomatic from a neurological perspective with no neurological deficits on physical exam. Therefore, no follow-up brain MRI was performed. MTX rechallenge was not attempted. He completed adjuvant chemotherapy with doxorubicin and cisplatin alone.

## Literature review results

We conducted a literature review using the University of Nebraska Medical Center McGoogan Health Sciences Library Catalog as a database and included all case reports of methotrexate-induced neurotoxicity in patients with osteosarcoma that were published from 2017 to 2022. Key words included: methotrexate, neurotoxicity, leukoencephalopathy, and osteosarcoma.

We present compiled data from 16 cases including ours, of MTX-induced neurotoxicity in patients with osteosarcoma. (Table 1) Ages ranged from 13- to 20-years-old. A total of 12 of the 16 patients (75%) were on cisplatin and doxorubicin chemotherapy regimens. All patients were administered IV MTX 12 g/m^2^ over 4 hours each cycle. The average number of MTX cycles was 4.17 cycles (1–11). The average number of days from last dose of MTX to day of MTX neurotoxicity presentation was 6.5 days (1–12). Time from symptom onset to improvement ranged from 30 minutes to 4 days. Time from symptom onset to full recovery ranged from 30 minutes to 9 days. Moreover, 12 of the 16 cases reported MTX blood level at symptom onset and in all cases MTX was at a nontoxic level.

**Table 1:** Compiled cases of methotrexate-induced neurotoxicity in patients with osteosarcoma

	Total *N* = 16 (%)
Demographics	
Gender	
Male, *n* (%)	12 (75%)
Female, *n* (%)	4 (25%)
Presentation	
General seizure	6 (37.5%)
Hemiparesis	7 (43.8%)
Dysarthria	5 (31.3%)
Dysphagia	2 (12.5%)
Altered mental status, impaired awareness, coma	7 (43.8%)
Paresthesia	1 (6.3%)
Methotrexate blood level	
Non-toxic blood level	12 (75%)
Not reported	4 (25%)
Leukoencephalopathy on brain MRI	
No	6 (37.5%)
Yes	9 (56.3%)
Unknown	1 (6.3%)
Treatments	
Dextromethorphan	
No	9 (56.3%)
Yes	7 (43.8%)
Aminophylline	
No	10 (62.5%)
Yes	6 (37.5%)
Ketamine	
No	15 (93.8%)
Yes	1 (6.3%)
Supportive treatment (dexamethasone, leucovorin)	
No	14 (87.5%)
Yes	2 (12.5%)
Any treatment	
No	7 (43.8%)
Yes	9 (56.3%)
No treatment	
No	9 (56.3%)
Yes	7 (43.8%)
Recurrence	
Recurrence of MTX neurotoxicity with subsequent cycles	
No recurrence with subsequent cycles	5 (31.3%)
No subsequent cycles	8 (50.0%)
Unknown	3 (18.8%)

*N* number, *MTX* methotrexate

The most common symptoms reported upon presentation were hemiparesis (6, 44%) and altered mental status (6, 44%). Nine (56%) had leukoencephalopathy on brain MRI and six (44%) had no changes on brain imaging.

Seven patients received no treatment for MTX-induced neurotoxicity and nine patients received single agent or combination treatment with leucovorin, steroids, dextromethorphan, aminophylline, or ketamine. Only one patient received ketamine. All patients fully recovered back to their original baseline. Subsequent MTX therapy was reportedly administered in 5 of 16 patients and no neurotoxicity was observed in any on rechallenge.

## Discussion

The pathogenesis of MTX-induced neurotoxicity is thought to involve central nervous system (CNS) folate homeostasis and/or direct neuronal damage [8]. There is no standard treatment for MTX-induced neurotoxicity and there are reports of full recovery without therapy. However, several nonFDA approved treatments including dextromethorphan (DM), aminophylline, and now ketamine have been documented in the literature [8–10].

MTX promotes the release of adenosine. Adenosine dilates cerebral blood vessels, modifies the release of pre- and postsynaptic neurotransmitters, and may slow the firing rate of neurons. Elevated adenosine has been demonstrated in cerebrospinal fluid (CSF) after MTX therapy. Aminophylline displaces adenosine from its receptor sites and is therefore thought to potentially improve adenosine neurotoxicity mediated by MTX [8]. Of note, there are currently no standardized dosing or monitoring recommendations for the off-label use of aminophylline in MTX-induced neurotoxicity. Monitoring theophylline (a metabolite of aminophylline) levels after administration of aminophylline is critical as toxic levels can be fatal. The therapeutic range for theophylline when used in the treatment of obstructive airway disease is 5–10 mcg/mL in children and 10–15 mcg/mL in adults. Toxic levels of theophylline occur at levels > 20 mcg/mL [11]. Therefore, we set the goal level of theophylline after aminophylline administration at 15–20 mcg/mL in our patient.

MTX inhibits the conversion of dihydrofolate to tetrahydro-folate and thus decreases the availability of 5-methyltetrahydrofolate (5-methyl-THF), which is a cofactor in the conversion of homocysteine to methionine. MTX-induced deficiency of 5-methyl THF therefore results in elevated homocysteine levels and decreased methionine levels [12]. Homocysteine has excitatory effects on the N-methyl-D-aspartate (NMDA) receptor, and also causes local and regional neuronal death by the activation of NADPH oxidase 2 (NOX2), which increases concentrations of superoxide and resultant oxidative stress. DM is used as treatment for MTX-induced neurotoxicity owing to its antagonistic effects on the NMDA receptor. [13]. One case report has proposed using ketamine as the agent of choice for patients with MTX-induced neurotoxicity who require sedation as ketamine is also a NMDA antagonist. Briefly, the patient was intubated owing to altered mental status and sedated with fentanyl and dexmedetomidine. Dextromethorphan was given for 2 days with no improvement. Fentanyl and dexmedetomidine infusions were then replaced with ketamine for sedation. After ketamine was initiated mental status began to improve and the patient was extubated [14].

MTX-related neurotoxicity is rare with a prevalence of 3.1–3.8% in patients with ALL [15–17]. Cumulative systemic MTX exposure, high methotrexate-to-leucovorin ratio, simultaneous use of intrathecal MTX and intravenous (IV) MTX, age greater than 10 years-old, higher MTX dose, and LatinX ethnicity have been implicated as risk factors for MTX neurotoxicity [8–10, 18]. Interestingly, MTX blood levels have not been shown to correlate with risk of neurotoxicity and blood levels are often within therapeutic range [19].

A multisite study of 280 pediatric patients with ALL found that MTX neurotoxicity occurred in 22% of LatinX compared with 7% of nonLatinX patients. A missense variant has been associated with susceptibility to MTX-induced neurotoxicity, and this variant was more common in LatinX (23%) compared with European (3%) or African (< 1%) populations [10].

Recurrence of neurotoxicity with MTX rechallenge is rare, although data on rechallenge is largely reported from ALL cases. [15, 19]. Bhojwani *et al* found that only 1 of 13 patients rechallenged with MTX had recurrence and some patients received up to 20 doses of IV MTX without developing any recurrence [15, 17]. Other studies have also reported a 0–1% recurrence rate in patients with ALL rechallenged with MTX [15, 20]. Several genome-wide association studies (GWAS) have identified single-nucleotide polymorphisms (SNPs) in the following genes: *TRIO, PRKG1, ANK1, COL4A2, NTN1*, and *ASTN2* that were associated with increased risk of MTX-induced neurotoxicity and leukoencephalopathy on imaging in patients with ALL. All of these genes play a role in neuronal development and migration [15, 17, 19]. These findings suggest that a similar genetic cause may be responsible for the recurrence of MTX neurotoxicity in certain patients, and the absence of recurrence in nearly all others. To the knowledge of these authors no other similar GWAS has been done on patients with osteosarcoma.

Given the low incidence of recurrence, most literature from patients with ALL supports resuming MTX after complete symptom resolution [21, 22]. However, there is very little data available on rechallenge with MTX in osteosarcoma. In addition, higher doses of MTX are used to treat osteosarcoma than ALL, making the decision to proceed with MTX rechallenge more daunting [23].

## Conclusion

Data on MTX-induced neurotoxicity largely comes from patients with ALL while data from patients with osteosarcoma is scarce. Our review adds to the limited existing literature on patients with osteosarcoma with MTX-induced neurotoxicity and can aid in the understanding of the complicated pathophysiology, diagnosis, and available treatments. Very few people who have experienced MTX neurotoxicity have recurrence on rechallenge. Further research is necessary to understand why only certain patients develop recurrence of neurotoxicity with rechallenge while others do not. In our review, 5 of 16 patients were known to be rechallenged with MTX. None had recurrence of neurotoxicity with subsequent MTX treatment. While our study is limited by the number of cases, our findings suggest that MTX rechallenge in patients with osteosarcoma could be considered. MTX is an important treatment for osteosarcoma and if safe, would be beneficial to continue even after acute neurotoxicity. Unfortunately, MTX is often discontinued after the first episode of neurotoxicity, due to fear of recurrence on rechallenge.

## Supplementary information

Supplementary material 1.

## Data Availability

Not applicable.

## Abbreviations

MTX: Methotrexate
ALL: Acute lymphoblastic leukemia
MRI: Magnetic resonance imaging
FLAIR: Fluid-attenuated inversion recovery
DWI: Diffusion weighted imaging
NMDA: N-methyl-d-aspartate
NOX2: NADPH oxidase 2
AMS: Altered mental status
CSF: Cerebrospinal fluid
FA: Folinic acid
DM: Dextromethorphan
