# Supplementary material for: Methotrexate-induced acute neurotoxicity in patients with osteosarcoma: a case report
Source: J Med Case Rep. 2025 Sep 30;19:473. doi: 10.1186/s13256-025-05500-y (PMC12487186; doi:10.1186/s13256-025-05500-y)
Supplement: Supplementary file 1 — Supplementary material 1. [file 13256_2025_5500_MOESM1_ESM.pdf]

CARE case report guidelines

CARE Checklist of information to include when writing a case report

(cc) BY-NC-ND

Topic	Item	Checklist item description	Reported on Line
**Title**	1	The diagnosis or intervention of primary focus followed by the words "case report"	Line 1
**Key Words**	2	2 to 5 key words that identify diagnoses or interventions in this case report, including "case report"	Line 63
**Abstract (no references)**	3a	Introduction: What is unique about this case and what does it add to the scientific literature?	Lines 38-42
	3b	Main symptoms and/or important clinical findings	Lines 38-39
	3c	The main diagnoses, therapeutic interventions, and outcomes	Lines 45-47
	3d	Conclusion—What is the main "take-away" lesson(s) from this case?	Lines 51-61
**Introduction**	4	One or two paragraphs summarizing why this case is unique (**may include references**)	Lines 106-113
**Patient Information**	5a	De-identified patient specific information.	Line 115
	5b	Primary concerns and symptoms of the patient.	Lines 116-117,128 and 135-137
	5c	Medical, family, and psycho-social history including relevant genetic information	Line 115
	5d	Relevant past interventions with outcomes	Lines117-127
**Clinical Findings**	6	Describe significant physical examination (PE) and important clinical findings.	Lines 135-137
**Timeline**	7	Historical and current information from this episode of care organized as a timeline	Lines 128-149
**Diagnostic Assessment**	8a	Diagnostic testing (such as PE, laboratory testing, imaging, surveys).	Lines 128-131, 137-140
	8b	Diagnostic challenges (such as access to testing, financial, or cultural)	N/A
	8c	Diagnosis (including other diagnoses considered)	Lines 133-135
	8d	Prognosis (such as staging in oncology) where applicable	Line 115
**Therapeutic Intervention**	9a	Types of therapeutic intervention (such as pharmacologic, surgical, preventive, self-care)	Lines 135,141-146
	9b	Administration of therapeutic intervention (such as dosage, strength, duration)	Lines 135, 141-146
	9c	Changes in therapeutic intervention (with rationale)	Lines 135, 141-146
**Follow-up and Outcomes**	10a	Clinician and patient-assessed outcomes (if available)	Lines 146-151
	10b	Important follow-up diagnostic and other test results	Lines 146-151
	10c	Intervention adherence and tolerability (How was this assessed?)	N/A
	10d	Adverse and unanticipated events	N/A
**Discussion**	11a	A scientific discussion of the strengths AND limitations associated with this case report	232-234
	11b	Discussion of the relevant medical literature **with references**.	Lines 170--249
	11c	The scientific rationale for any conclusions (including assessment of possible causes)	Lines 170-249
	11d	The primary "take-away" lessons of this case report (without references) in a one paragraph conclusion	Lines 251-261
**Patient Perspective**	12	The patient should share their perspective in one to two paragraphs on the treatment(s) they received	N/A
**Informed Consent**	13	Did the patient give informed consent? Please provide if requested	Yes ☑ No ☐
